# Synergic Heterodinuclear Catalysts for the Ring-Opening Copolymerization (ROCOP) of Epoxides, Carbon Dioxide, and Anhydrides

**DOI:** 10.1021/acs.accounts.2c00197

**Published:** 2022-07-21

**Authors:** Wilfred T. Diment, Wouter Lindeboom, Francesca Fiorentini, Arron C. Deacy, Charlotte K. Williams

**Affiliations:** Chemistry Research Laboratory, Department of Chemistry, University of Oxford, 12 Mansfield Road, Oxford OX1 3TA, United Kingdom

## Abstract

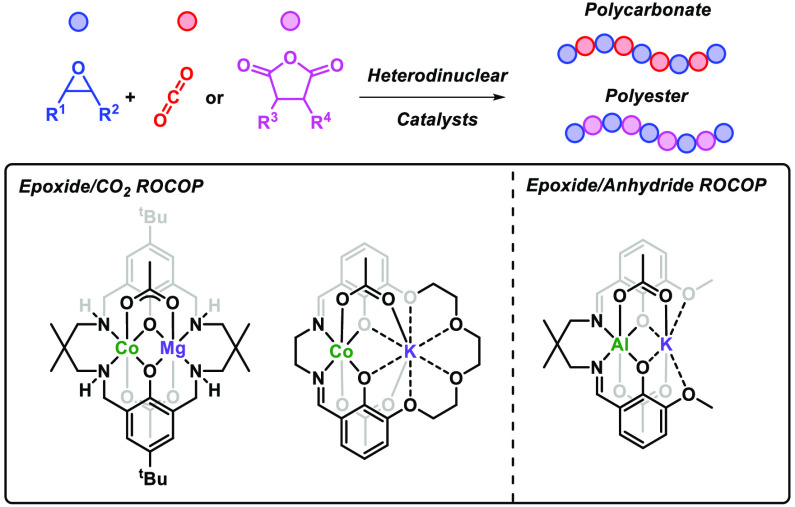

The development of sustainable plastic materials is an essential target of chemistry in the 21st century. Key objectives toward this goal include utilizing sustainable monomers and the development of polymers that can be chemically recycled/degraded. Polycarbonates synthesized from the ring-opening copolymerization (ROCOP) of epoxides and CO_2_, and polyesters synthesized from the ROCOP of epoxides and anhydrides, meet these criteria. Despite this, designing efficient catalysts for these processes remains challenging. Typical issues include the requirement for high catalyst loading; low catalytic activities in comparison with other commercialized polymerizations; and the requirement of costly, toxic cocatalysts. The development of efficient catalysts for both types of ROCOP is highly desirable. This Account details our work on the development of catalysts for these two related polymerizations and, in particular, focuses on dinuclear complexes, which are typically applied without any cocatalyst. We have developed mechanistic hypotheses in tandem with our catalysts, and throughout the Account, we describe the kinetic, computational, and structure–activity studies that underpin the performance of these catalysts. Our initial research on homodinuclear M(II)M(II) complexes for cyclohexene oxide (CHO)/CO_2_ ROCOP provided data to support a chain shuttling catalytic mechanism, which implied different roles for the two metals in the catalysis. This mechanistic hypothesis inspired the development of mixed-metal, heterodinuclear catalysts. The first of this class of catalysts was a heterodinuclear Zn(II)Mg(II) complex, which showed higher rates than either of the homodinuclear [Zn(II)Zn(II) and Mg(II)Mg(II)] analogues for CHO/CO_2_ ROCOP. Expanding on this finding, we subsequently developed a Co(II)Mg(II) complex that showed field leading rates for CHO/CO_2_ ROCOP and allowed for unique insight into the role of the two metals in this complex, where it was established that the Mg(II) center reduced transition state entropy and the Co(II) center reduced transition state enthalpy. Following these discoveries, we subsequently developed a range of heterodinuclear M(III)M(I) catalysts that were capable of catalyzing a broad range of copolymerizations, including the ring-opening copolymerization of CHO/CO_2_, propylene oxide (PO)/CO_2_, and CHO/phthalic anhydride (PA). Catalysts featuring Co(III)K(I) and Al(III)K(I) were found to be exceptionally effective for PO/CO_2_ and CHO/PA ROCOP, respectively. Such M(III)M(I) complexes operate through a dinuclear metalate mechanism, where the M(III) binds and activates monomers while the M(I) species binds the polymer change in close proximity to allow for insertion into the activated monomer. Our research illustrates how careful catalyst design can yield highly efficient systems and how the development of mechanistic understanding aids this process. Avenues of future research are also discussed, including the applicability of these heterodinuclear catalysts in the synthesis of sustainable materials.

## Key References

- KemberM. R.; KnightP. D.; ReungP. T. R.; WilliamsC. K.Highly Active Dizinc Catalyst for the Copolymerization of Carbon Dioxide and Cyclohexene Oxide at One Atmosphere Pressure. Angew. Chem. Int. Ed., 2009, 48, 931–93310.1002/anie.20080389619115338.^1^
  *A highly active Zn(II)Zn(II) complex, coordinated by a macrocyclic ligand, for cyclohexene oxide/CO*_*2*_*ring-opening copolymerization (ROCOP); the catalyst turns over at low CO*_*2*_*pressure (1 bar).*
- DeacyA. C.; KilpatrickA. F. R.; RegoutzA.; WilliamsC. K.Understanding metal synergy in heterodinuclear catalysts for the copolymerization of CO_2_ and epoxides. Nat. Chem., 2020, 12, 372–3803222150110.1038/s41557-020-0450-3.^2^
  *A highly active heterodinuclear Mg(II)Co(II) catalyst for cyclohexene oxide/CO*_*2*_*ROCOP. Kinetic investigations reveal that synergy depends upon different roles for each metal.*
- DeacyA. C.; MorebyE.; PhanopoulosA.; WilliamsC. K.Co(III)/Alkali-Metal(I) Heterodinuclear Catalysts for the Ring-Opening Copolymerization of CO_2_ and Propylene Oxide. J. Am. Chem. Soc., 2020, 142, 19150–191603310873610.1021/jacs.0c07980PMC7662907.^3^
  *A series of heterodinuclear catalysts, Co(III)M(I), coordinated by a different macrocyclic ligand, for propylene oxide/CO*_*2*_*ROCOP. The Co(III)K(I) catalyst shows both high activities and selectivities and it operates with chain transfer agents and a range of epoxide monomers.*
- DimentW. T.; GregoryG. L.; KerrR. W. F.; PhanopoulosA.; BuchardA.; WilliamsC. K.Catalytic Synergy Using Al(III) and Group 1 Metals to Accelerate Epoxide and Anhydride Ring-Opening Copolymerizations. ACS Catal.2021, 11, 12532–12542.^4^
  *A series of heterodinuclear Al(III)M(I) catalysts, coordinated by a Schiff base ligand, for epoxide/anhydride ROCOP. The Al(III)K(I) catalyst shows exceptional activities, loading tolerance, and broad monomer scope. Kinetic and computational calculations reveal a mechanism operating via a metalate intermediate.*

## Introduction

**Scheme 1:**
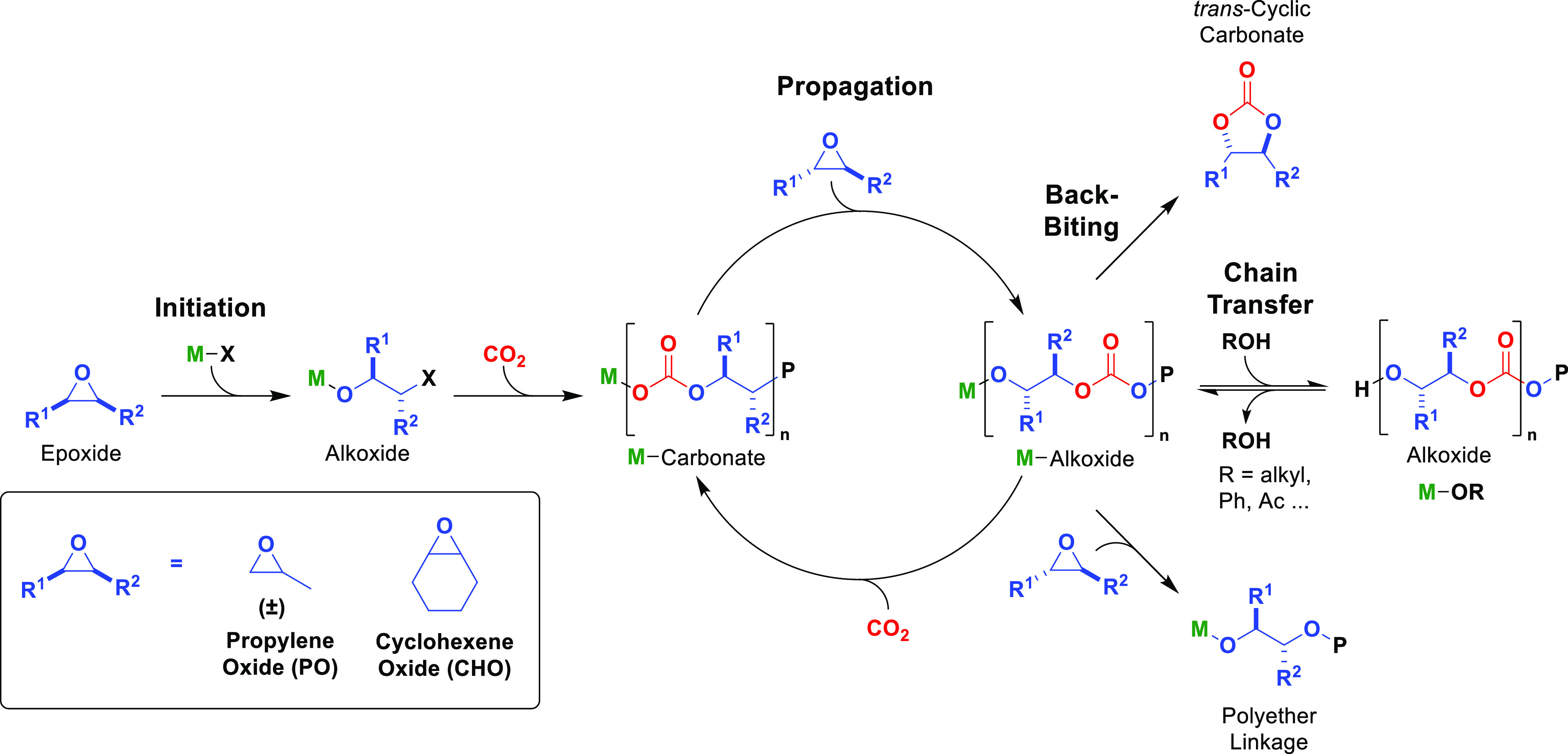
Summary of Ring-Opening Copolymerization (ROCOP) for Epoxide/CO_2_ An analogous mechanism is followed for epoxide/anhydride ROCOP.

The efficient conversion of carbon dioxide to useful products is a linchpin of sustainable chemistry. Carbon dioxide is an abundant and low-cost C_1_ feedstock and is a waste product of many industrial processes, including combustion and fermentation.^5^ One promising avenue for its valorization and recycling is to produce polycarbonates via the ring-opening copolymerization (ROCOP) of carbon dioxide with epoxides (Scheme 1).^6^ The related ROCOP of epoxides and anhydrides is a broadly applicable and controlled process delivering polyesters. In both reactions, the polymerization catalyst is essential for cost-effective manufacturing and to control the polymers’ properties.

This Account describes some of our discoveries, developments, and growing understanding of dinuclear ring-opening copolymerization catalysts. Prior to presenting such a personal perspective, we would like to acknowledge the pioneering initial discoveries of R.F. Fischer of Shell Development Company (epoxide/anhydride ROCOP) and Shohei Inoue of the University of Tokyo (carbon dioxide/epoxide ROCOP).^7,8^ This field of polymerization catalysis has since benefited from the innovation and creativity of many other groups worldwide, including those of Kuran, Darensbourg, Nozaki, Coates, Lu, Lee, Wang, Koning, Gürtler, Hadjichristidis, Gnanou, Chisholm, Kleij, Frey, Grinstaff, Reiger, Leitner, Wu, Kozak, Luinstra, Ree, and many more. Our Account cannot describe all their innovations and influences; rather, the reader is directed to some excellent comprehensive reviews.^6,9−13^

Before designing a new catalyst, it is essential to consider the polymerizations’ elementary steps. Epoxide/carbon dioxide ROCOP involves this series of reactions: initiation, propagation, and chain transfer, with backbiting and polyether formation both possible as side-reactions. Epoxide/anhydride ROCOP follows an analogous mechanism with CO_2_ replaced by anhydride (Scheme 1).^6^ During initiation, an activated epoxide molecule is ring-opened by a nucleophile or initiating group, **X** (typically an acetate, alkoxide, halide, or carboxylate), which generates a metal alkoxide intermediate. Subsequent, rapid insertion of CO_2_ into the metal alkoxide generates a metal–carbonate intermediate and starts the propagation catalytic cycle. During propagation, the carbonate intermediate ring-opens another activated epoxide to (re)form a metal alkoxide, which then inserts CO_2_ to (re)form the carbonate; multiple repetitions build up the polymer.

We target controlled polymerization catalysts, meaning that the polymer molar mass values are predictable, evolve linearly, and show narrow molar mass distributions (*Đ* < 1.2). These are not strictly *living* polymerizations, but are sometimes described as “*immortal*,” because chain transfer reactions are endemic (Scheme 1).^14^ They involve a rapid exchange of metal alkoxide intermediates with protic species that are either present in or added to the reaction. Common chain transfer agents (CTAs) are alcohols, and the exchange (transfer) reactions typically occur faster than propagation and therefore do not influence polymerization kinetics.^15^ Rather, chain transfer controls molar mass and chain end-group chemistry without increasing the quantity of catalyst. Although this Account principally focuses on the activity and selectivity of our catalysts, it should be noted that in all cases our catalysts display good reaction control (*Đ* < 1.2) and immortal polymerization behavior, which allows for controllable molar mass polymers from polyols (<5 kg mol^–1^) up to higher molar mass samples (50 kg mol^–1^) depending on the amount of CTA applied to the polymerizations.

In carbon dioxide/epoxide ROCOP, the major side product is *trans*-cyclic carbonate, which is usually formed by intramolecular reaction (backbiting) of the metal alkoxide with the polymer chain (Scheme 1). Another side-reaction is the metal alkoxide reaction with epoxide to form (poly)ether linkages. This process decreases the overall CO_2_ uptake but can be useful to moderate polymers’ thermal-mechanical properties.^16^ In epoxide/anhydride ROCOP, in contrast to epoxide/CO_2_ ROCOP, backbiting reactions usually have higher energy barriers, so cyclic side products (lactones) are not usually observed; however, polyether formation can still be observed as a side reaction.

Effective polymerization catalysts show high productivity [turnover number (TON)] and activity [turnover frequency (TOF)] at minimal catalyst loadings and, for reactions involving CO_2_, at pressures of <10 bar, which allows for the retrofitting of current industrial plants. They deliver polymers with predictable molar mass, monomer sequence (% carbonate/ester selectivity), end-group chemistry, and minimal side products (% polymer selectivity). They should also be inexpensive, lightweight, straightforward to synthesize, nontoxic, redox inactive, and removable/recoverable after use. Our team has investigated dinuclear catalysts comprising light main-group (particularly s-block) and first row transition metal elements. These complexes apply ancillary ligands featuring phenolate and amines/imines since these “hard” donors form stable, activated complexes. The internuclear separations of these complexes are in the range of 3–4 Å, which enables intermetallic electronic communication and avoids the need for an addition of cocatalyst, the use of which can limit activity at low catalyst loading.

**Figure 1:**
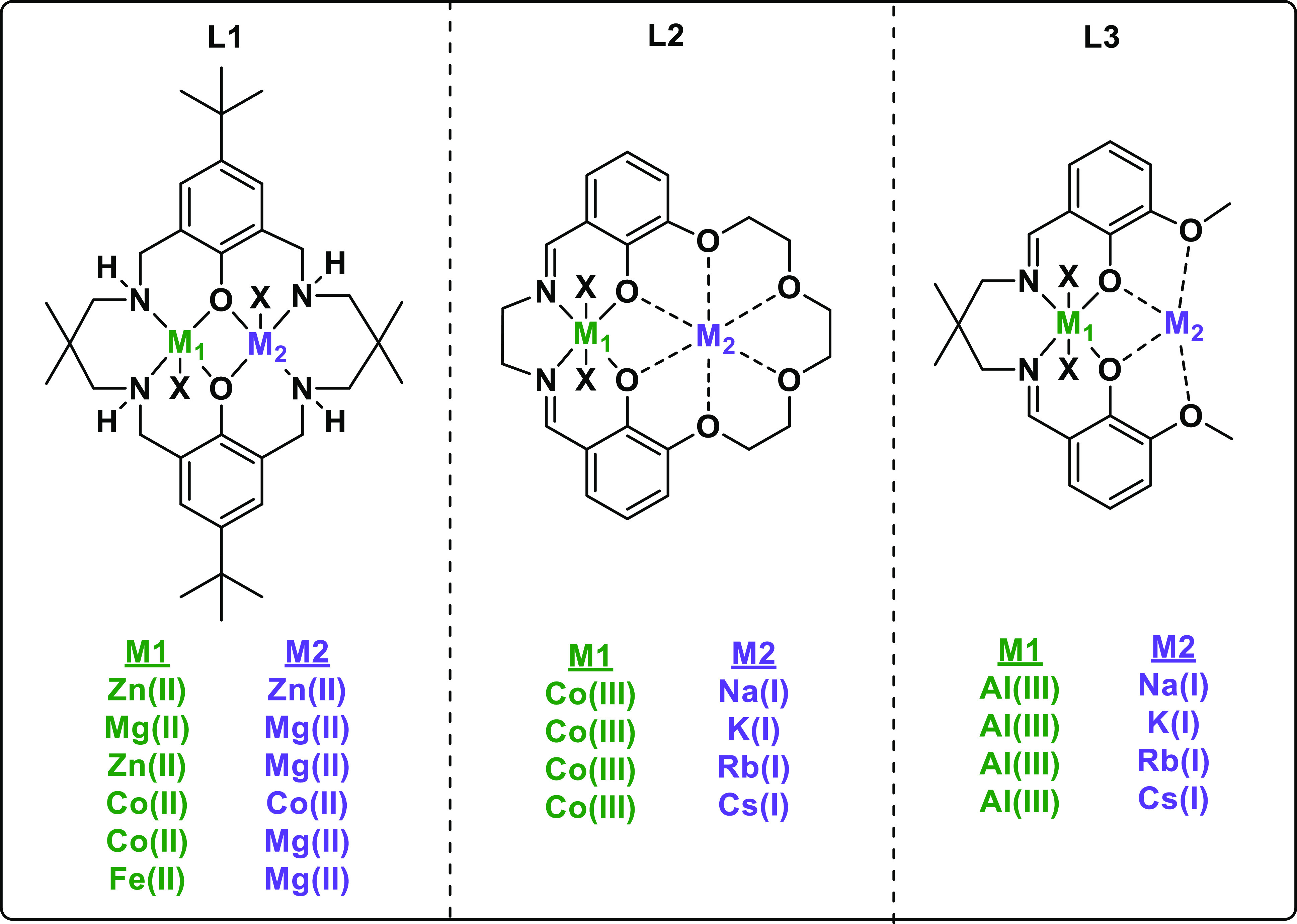
Ligands and some of the key metal combinations used in dinuclear catalysts for epoxide/CO_2_ and epoxide/anhydride ROCOP. X = anionic coligand (typically acetate, halide, or benzoate).

Our initial hypothesis was that dinuclear catalysts should show lower barriers to epoxide ring-opening and heteroallene insertion reactions compared with mononuclear active sites. It was inspired by nature’s bimetallic metalloenzymes, e.g., di-Zn(II) hydrolases, phosphatases, or peptidases, which follow mechanisms involving dinuclear substrate activation and nucleophilic attack steps (often with water).^17,18^ It was also underpinned by outstanding work from other researchers in the field investigating di- or multinuclear catalysts. Since the late 1970s, there were reports of multinuclear Zn(II) catalysts,^19^ and particular mention is warranted of Zn(II)phenoxides from Darensbourg et al., Zn(II) β-diimine (BDI) dimeric catalysts from Coates et al., anilido aniline di-Zn(II) catalysts from Lee et al., and di-Zn(II) complexes from Nozaki et al.^20−24^ The selection of the ancillary ligand is critical to control both catalytic stability and performance. Our approach has been to explore dinucleating Schiff base ligands; this Account focuses on three of them: **L1** for cyclohexene oxide (CHO)/CO_2_; **L2** for propylene oxide (PO)/CO_2_; and **L3** for CHO/phthalic anhydride (PA) (Figure 1). Henceforth, research using these ligands is described, and for efficiency, complexes are referred to as M_1_(oxidation state)M_2_(oxidation state).

### Epoxide/CO_2_ ROCOP with **L1**

In 2009, we reported a Zn(II)Zn(II) complex, using **L1** as an ancillary ligand, that was active for the copolymerization of carbon dioxide with CHO under 1 bar of pressure.^1^ It showed a TOF of 25 h^–1^ when using 0.1 mol % catalyst at 100 °C (1:1000, catalyst/CHO), even when using unoptimized conditions, i.e., a glass Schlenk line and magnetic stirring. It also showed quantitative carbon dioxide uptake and high polymer selectivity; it was a rare example of a ROCOP catalyst active at low CO_2_ pressure. The polymer product, poly(cyclohexene carbonate) (PCHC), shows a high glass transition temperature (*T*_g_) (105– 115 °C), tensile strength (42 MPa), and Young’s modulus (3.6 GPa).^25^

The macrocyclic ancillary ligand was also very effective in coordinating a range of s-block and first row transition metals with a metal–metal distance of ∼3.0 Å, as confirmed by single crystal X-ray diffraction.^26−30^ Our subsequent reports described a range of homodinuclear catalysts, in some cases as the first examples of catalysts using those metals/oxidation states: Mg(II)Mg(II),^31^ Fe(III)Fe(III),^27^ Co(II)Co(II), and Co(II)Co(III).^26,28^ We were particularly interested in the Mg(II)Mg(II) catalyst, which was twice as active as the Zn(II)Zn(II) catalyst, yet Mg(II) is 1/3 of Zn(II)’s mass and also shows 2000 times higher elemental abundance.

These dinuclear catalysts showed unparalleled activity using low-pressure carbon dioxide sources, thereby obviating the use of specialized high-pressure steel equipment, and are tolerant of large amounts of CTA, including water. These features are attractive for the coupling of carbon dioxide use with captured emissions. At the time of the study, the UK operated an amine-based carbon capture and storage demonstrator plant at Ferrybridge, a coal-fired power station, and we were able to source captured carbon dioxide emissions from the plant in a Tedlar bag. Both the Zn(II)Zn(II) and Mg(II)Mg(II) catalysts showed impressive performances using this impure carbon dioxide gas, even under unoptimized conditions, i.e., using heavy textbooks as weights to force the gas from the Tedlar bag into the Schlenk tube. We also investigated the effects of deliberately added impurities to the carbon dioxide: the catalysts remained active using carbon dioxide contaminated by high concentrations of water, carbon monoxide, nitrogen, oxygen, amines, and thiols. The study demonstrates the potential to couple carbon dioxide utilization with large-scale capture and storage technologies.^32^

**Figure 2:**
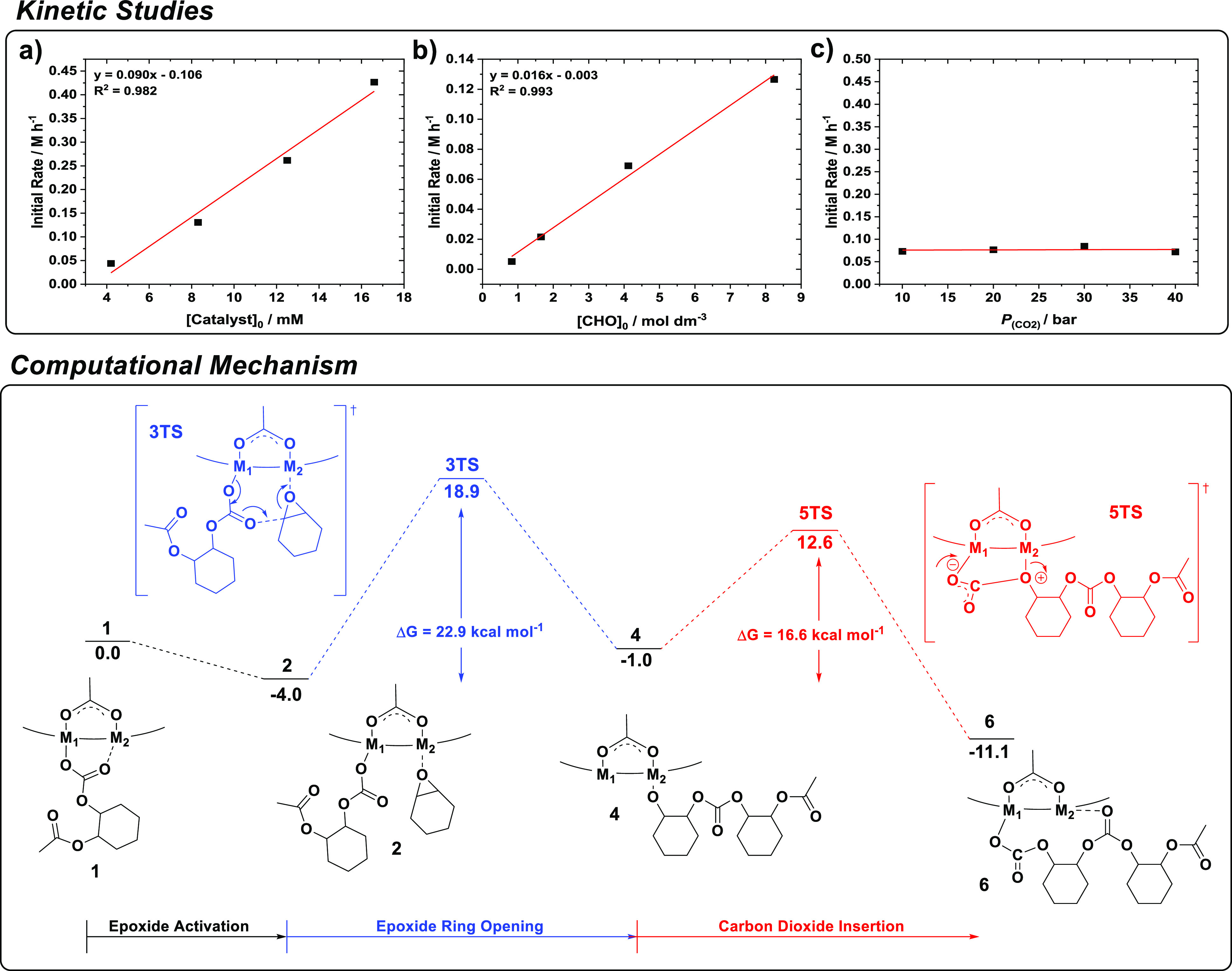
Data for CHO/CO_2_ ROCOP kinetics and its proposed mechanism using Zn(II)Zn(II). Kinetic studies, top panel: (a) plot of initial rate versus catalyst concentration, which indicates a first-order dependence; (b) plot of initial rate versus epoxide concentration, which indicates a first-order dependence; (c) plot of initial rate versus carbon dioxide pressure, which indicates zero-order dependence. All images adapted with permission from ref (33). Copyright 2011 American Chemical Society. Computational mechanism, bottom panel: the potential energy surface, determined by DFT calculations [theory level: ωB97XD/6-31G(d)], for the alternating copolymerization.

A detailed kinetic investigation including elucidation of the polymerization rate law using the Zn(II)Zn(II) catalyst was undertaken to understand the performances of these dinuclear catalysts (Figure 2, top).^33^ The copolymerization shows first-order rate dependencies in both the catalyst and epoxide concentrations, but is zero-order in the carbon dioxide pressure (1–40 bar). The rate-limiting step was proposed as CHO ring-opening by the Zn-carbonate intermediate.

In collaboration with Henry Rzepa at Imperial College London, a DFT investigation of the polymerization mechanism examined all possible permutations of four monomer insertions; the calculations accounted for the polar reaction medium (neat epoxide) and temperature (Figure 1, bottom).^34^ The study suggests that initiation occurs from Zn(II)Zn(II) featuring two bridging acetate ligands; in situ IR spectroscopy supported this speciation (**1**). CHO coordination at Zn(II) (M_2_) (**2**) is slightly exothermic (−4.0 kcal mol^–1^) and is followed by nucleophilic attack of a Zn-acetate (or M_1_-carbonate) (3TS) to form the Zn-alkoxide (M_2_) (**4**). In situ IR spectroscopy suggests that only one of the two acetate groups initiates, while the other remains coordinated and counterbalances the change in coordination chemistry of the growing polymer chain. Carbon dioxide insertion proceeds via initial ligand activation and generation of a zwitterionic intermediate, which rearranges (**5TS**) to a Zn-carbonate (M_1_) species (**6**). The calculations show that CHO ring-opening is the rate-determining step, with a transition state barrier of 22.9 kcal mol^–1^, compared with a lower barrier to carbon dioxide insertion (16.6 kcal mol^–1^). The DFT barrier for the rate-limiting step is close to the value determined experimentally (25.7 kcal mol^–1^), thereby giving confidence in the mechanism.^33^ The mechanism shows that the growing polymer chain exchanges twice between the two metal centers per catalytic cycle; it was, therefore, termed the “chain shuttling mechanism.”^34^ The movement of the growing polymer chain between metal sites is facilitated by the bridging acetate ligand; in situ IR studies reveal both Zn-coordinated and polymer chain end-functionalized acetate groups during propagation. The mechanism shows different roles for the two Zn(II) centers (hence the use of M_1_ and M_2_): (1) epoxide coordination and activation and/or (2) provision of a labile, nucleophilic carbonate group. The chain shuttling mechanism inspired the investigation of heterodinuclear metal complexes, i.e., where M_1_ differs from M_2_.

The initial investigation targeted Mg(II)Zn(II) catalysts because of the precedent for the homodinuclear catalysts, the ability to monitor complexation using ^1^H NMR spectroscopy, and the rate enhancement shown by Mg(II)Mg(II).^35^ The initial complex synthesis involved adding each metal precursor sequentially to the macrocycle at room temperature. It formed a 1:2:1 mixture of Zn(II)Zn(II), Mg(II)Zn(II), and Mg(II)Mg(II), as determined by both ^1^H NMR spectroscopy and mass spectrometry. The statistical mixture of catalysts showed higher rates, under identical conditions, than either of the homodinuclear catalysts or a 1:1 mixture of the two catalysts. These results suggested some intermetallic synergy and motivated the isolation of a pure heterodinuclear catalyst.

Adapting the synthetic procedure allowed isolation of a pure heterodinuclear Mg(II)Zn(II) catalyst.^36^ The Mg(II)Zn(II) catalyst showed a TOF of 34 h^–1^ (1 bar of CO_2_, 80 °C) and was twice the activity of Mg(II)Mg(II) and 5 times faster than a 1:1 mixture of Mg(II)Mg(II) and Zn(II)Zn(II) (Table 1, entries 1–3).

**Table 1:** Homo- and Heterodinuclear CHO/CO_2_ ROCOP Catalysts Using **L1**^a^

#	catalyst	time (h)	conv.^b^(%)	CO_2_^c^ (%)	polym.^d^ (%)	TON^e^	TOF^f^ (h^–1^)	*M*_n_ [*Đ*]^g^ (g mol^–1^)
1	Mg(II)Mg(II)	10	15	>99	>99	151	15	800 [1.13]
2	Mg(II)Zn(II)	10	34	>99	>99	344	34	3100 [1.14]
3	Mg(II)Mg(II)/Zn(II)Zn(II)(1:1)	10	7	>99	>99	72	7	<500
4^h^	Mg(II)Zn(II)	3	37	>99	>99	372	124	10 200 [1.02] 4750 [1.08]
5^i^	Mg(II)Zn(II)	0.5	44	>99	>99	4415	8830	44 400 [1.04] 21 200 [1.05]

a Reaction conditions: [Cat]/[CHO] = 1:1000, neat epoxide, 1 bar CO_2_, 80 °C.^36^ Note that under these conditions, the Zn(II)Zn(II) did not initiate polymerization, as the study utilized bromide coligands.

b Conversion of epoxide, determined by ^1^H NMR.

c Selectivity for carbonate vs ether, determined by ^1^H NMR.

d Selectivity for polymer vs cyclic carbonate, determined by ^1^H NMR.

e Turnover number (TON) = total number of moles of epoxide consumed/mol of catalyst.

f Turnover frequency (TOF) = TON/time (hours).

g Number average molecular weight [dispersity], determined by GPC.

h Conditions: [Cat]/[CHO] = 1:1000, 1 bar CO_2_, 80 °C.^37^

i Conditions: [Cat]/[CHO] = 1:10000, 20 bar CO_2_, 120 °C.^37^

Subsequent optimization of the catalyst coligands and of the reaction conditions yielded increases in catalytic activities.^37^ At 1 bar of CO_2_ pressure and 80 °C, a Mg(II)Zn(II) catalyst bearing nitro-benzoate coligands gave a TOF of 124 h^–1^ (Table 1, entry 4). The same catalyst yielded a TOF of 8830 h^–1^ when CO_2_ pressure was increased to 20 bar, the temperature was increased to 120 °C, and the reaction was performed with impeller stirring (Table 1, entry 5).

Following this initial study, in order to better understand which metal combinations were synergic, Zn(II) was combined with other alkali [Li(I), Na(I), K(I)], alkaline earth [Ca(II)], and Group 13 [Al(III), Ga(III), In(III)] metals to form heterodinuclear complexes.^29,30^ This work sheds light upon synthesis procedures: reactions were conducted either under kinetic (−78 °C) or thermodynamic (100 °C) control in the second metalation step. All the reactions resulted in quantitative heterodinuclear complex formation (>95% by NMR spectroscopy), and almost all the complexes, except Zn(II)Na(I), are thermodynamically more stable than their homodinuclear analogues. The high selectivity and stability for heterodinuclear complexes was at first quite surprising because of the symmetrical macrocycle with identical metal coordination sites, but is attributed to the additional stability arising from polarized M_1_–O–M_2_ interactions enabled by the phenoxide bridges. Nonetheless, in nearly all the cases, the heterodinuclear complexes were less active than the Zn(II)Zn(II) catalyst, with the exception being the synergic Zn(II)Mg(II) catalyst.

**Figure 3:**
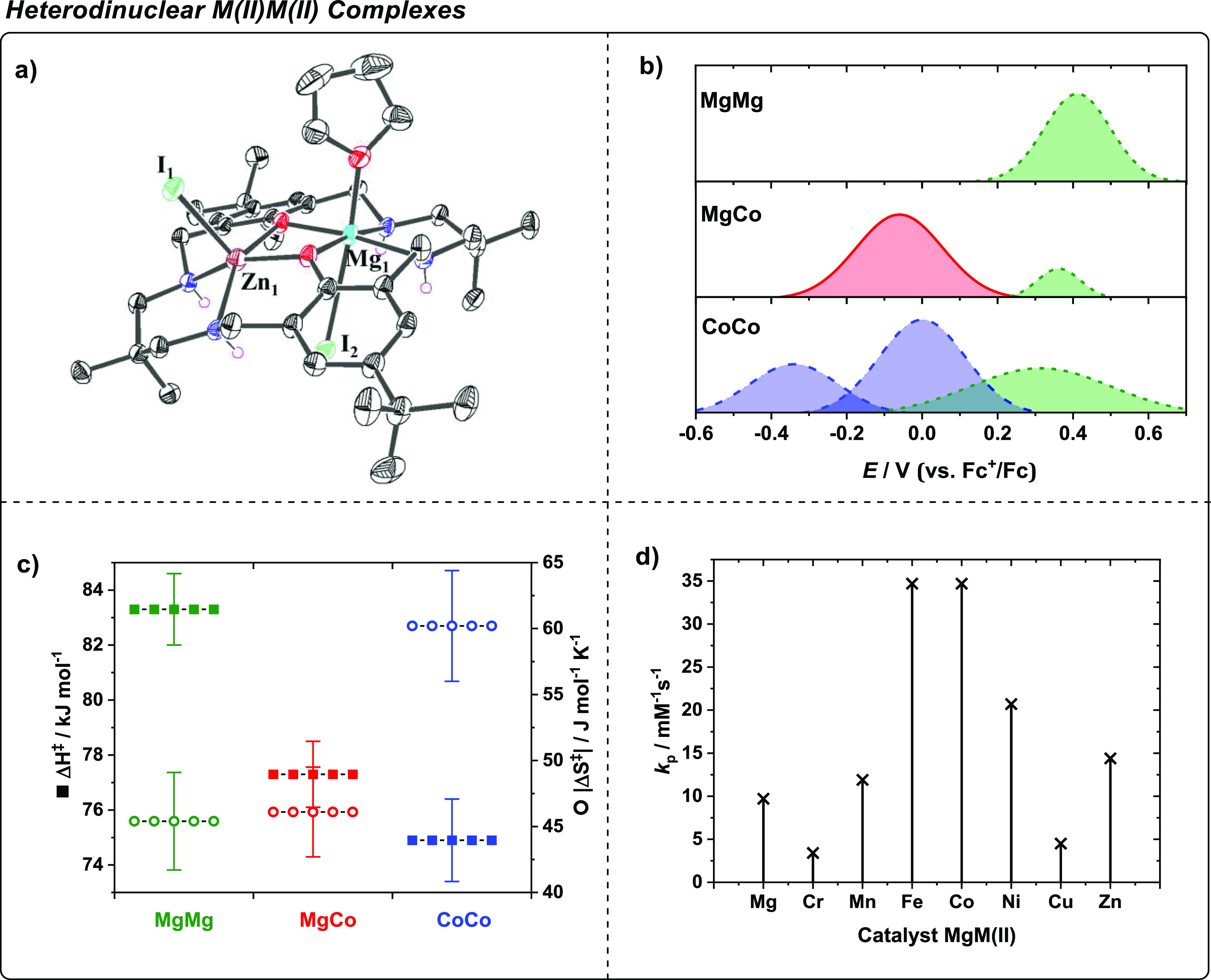
Data for heterodinuclear M(II)M(II) complexes applied in CHO/CO_2_ ROCOP. (a) Molecular structure of a Zn(II)Mg(II) complex showing THF bound to the Mg(II) center as a mimic for epoxide coordination. Image adopted with permission from ref (29). Copyright 2018 American Chemical Society. (b) Cyclic voltammetry plots showing oxidations and establishing the Mg(II)Co(II) complex. Image adopted with permission from ref (2). Copyright 2020 Springer Nature. (c) Plot comparing transition state enthalpy (Δ*H*^‡^) and entropy (Δ*S*^‡^) barriers for Mg(II)Mg(II), Co(II)Co(II), and Mg(II)Co(II), which indicates the basis for heterodinuclear synergy. Image adopted with permission from ref (2). Copyright 2020 Springer Nature. (d) Plot showing the variation in propagation rate constants (*k*_p_) for various heterodinuclear complexes, Mg(II)M(II), in CHO/CO_2_ ROCOP. Image adopted with permission from ref (38). Copyright 2022 Wiley.

Rationalizing the lack of synergy is, of course, challenging but may arise from factors such as saturated metal coordination spheres (Group 13), large ionic radii metals preventing planar coordination [Ca(II)], or small ionic radii metals forming heterodinuclear aggregates [Li(I)]. A molecular structure of a Mg(II)Zn(II) precatalyst, determined by single crystal X-ray diffraction, showed THF coordination at Mg(II), which may well be relevant to epoxide binding during catalysis (Figure 3a).

Following the investigation of Zn(II)-main group element combinations, we targeted Mg(II)M′(II) complexes, where M′ is a first row transition metal. Some years earlier, we reported a Co(II)Co(II) catalyst that showed good activity, and understanding whether synergic effects could boost its performance was a priority. Thus, the Mg(II)Co(II) catalyst was synthesized using the sequential metalation approach; quantifying the heterodinuclear complex formation was challenging because of the paramagnetism of Co(II).^2^ The complex was characterized using SQUID, cyclic voltammetry, and XPS; all techniques indicated a pure heterodinuclear complex formation. For example, Co(II)Co(II) shows two cobalt-centered oxidations, while Mg(II)Mg(II) shows only an irreversible, high-energy ligand oxidation, and Mg(II)Co(II) displays only one metal oxidation (Figure 3b).

Under 1 bar of CO_2_ pressure and at 120 °C, Mg(II)Co(II) is significantly faster than Mg(II)Mg(II), Co(II)Co(II), or physical mixtures of both (Table 2, entries 1–3). It showed a maximum activity of 1205 h^–1^, maintained quantitative CO_2_ uptake and polymer selectivity, and is at the forefront of low-pressure catalysts in the field.

**Table 2:** Homo- and Heterodinuclear CHO/CO_2_ ROCOP Catalysts Using **L1**^a^

#	catalyst	*p*(CO_2_) (bar)	conv.^b^ (%)	CO_2_^c^ (%)	polym.^d^ (%)	TON^e^	TOF (h^–1^)^f^
1	Mg(II)Mg(II)	1	18	>99	>99	368	368
2	Co(II)Co(II)	1	36	>99	96	712	712
3	Mg(II)Co(II)	1	25	>99	99	502	1205
4	Mg(II)Mg(II)	20	91	>99	>99	1820	1060
5	Co(II)Co(II)	20	94	>99	>99	1880	4200
6	Mg(II)Co(II)	20	95	>99	>99	1900	7200
7^g^	Mg(II)Co(II)	20	96	>99	>99	1920	12460

a Reaction conditions: [Cat]/[*trans*-1,2-cyclohexane diol (CHD)]/[CHO] = 1:20:2000, neat epoxide, 120 °C.

b Conversion of epoxide, determined by ^1^H NMR.

c Selectivity for carbonate vs ether, determined by ^1^H NMR.

d Selectivity for polymer vs cyclic carbonate, determined by ^1^H NMR.

e Turnover number (TON) = total number of moles of epoxide consumed/mol of catalyst.

f Turnover frequency (TOF) = TON/time (hours).

g [Cat]/[CHD]/[CHO] = 1:20:2000, 3 M CHO in diethylcarbonate, 140 °C.^2^

Increasing the CO_2_ pressure (20 bar) while maintaining a temperature of 120 °C significantly increased the rate, primarily because of the enhanced mechanical stirring in high pressure equipment, which overcame the diffusion limitations at low pressure (Table 2, entries 4–6). Such high-pressure polymerizations can also be conducted at higher temperature (140 °C) without forming any cyclic carbonate side products. Under such conditions, a very high TOF of 12 460 h^–1^ was observed: this remains the most active catalyst yet reported for perfectly alternating PCHC (Table 2, entry 7). Further, the impressive activity was maintained under high dilution, and conditions were optimized to allow for complete epoxide conversion (TON = 1920).

Undertaking polymerization kinetic analyses at different temperatures enabled determination of the transition state enthalpy (Δ*H*^‡^) and entropy (Δ*S*^‡^) barriers (Figure 3c). Comparing the barriers for the homo- vs heterodinuclear catalysts revealed that Co(II)Co(II) has a lower transition state enthalpy and that Mg(II)Mg(II) has a lower entropy barrier. Accordingly, Mg(II)Co(II) shows the best of both metals, with the Co(II) center reducing the reaction enthalpy, and the Mg(II) center reducing the reaction entropy. The data imply that each metal has a distinctive role in catalysis and that the bridging phenolate groups help moderate the active site electronics. The rate-determining step involves CHO coordination at Mg(II), which is attacked by the Co(II)-carbonate, and the reaction follows the chain shuttling mechanism.

The synergic combination of s-block Mg(II) and Co(II) motivated the investigation of other Mg(II) transition-metal(II) catalysts.^38^ All transition metals were applied in the +2 oxidation state to (1) allow for a direct comparison with Mg(II)Co(II) and Mg(II)Zn(II) catalysts and (2) ensure all catalysts applied commercial M(OAc)_2_ precursors; hence, the metals featured common initiating groups. The catalysts were synthesized using the sequential metalation route, whereby Mg(II) was first coordinated by the macrocycle, followed by the addition of the second transition metal and heating at 100 °C for 16 h. The complexes were characterized by mass spectrometry, IR spectroscopy, and cyclic voltammetry. All the heterodinuclear complexes were catalysts for the ring-opening copolymerization of carbon dioxide with CHO and most were more active than Mg(II)Mg(II). The Mg(II)Mn(II) catalyst was highly active, which was unexpected since prior research showed that Mn(III) porphyrin or salen complexes are poorly active.^39^ The Mg(II)Fe(II) catalyst displayed exceptional activity, equivalent to that obtained for the Mg(II)Co(II) catalyst, and is significant because iron is ∼3000 times more abundant, cheaper, and less toxic than cobalt. The series of complexes show a “volcano” type activity profile: the best performances arise using midrow transition metals (Figure 3d).

### Epoxide/CO_2_ ROCOP using **L2**

**Figure 4:**
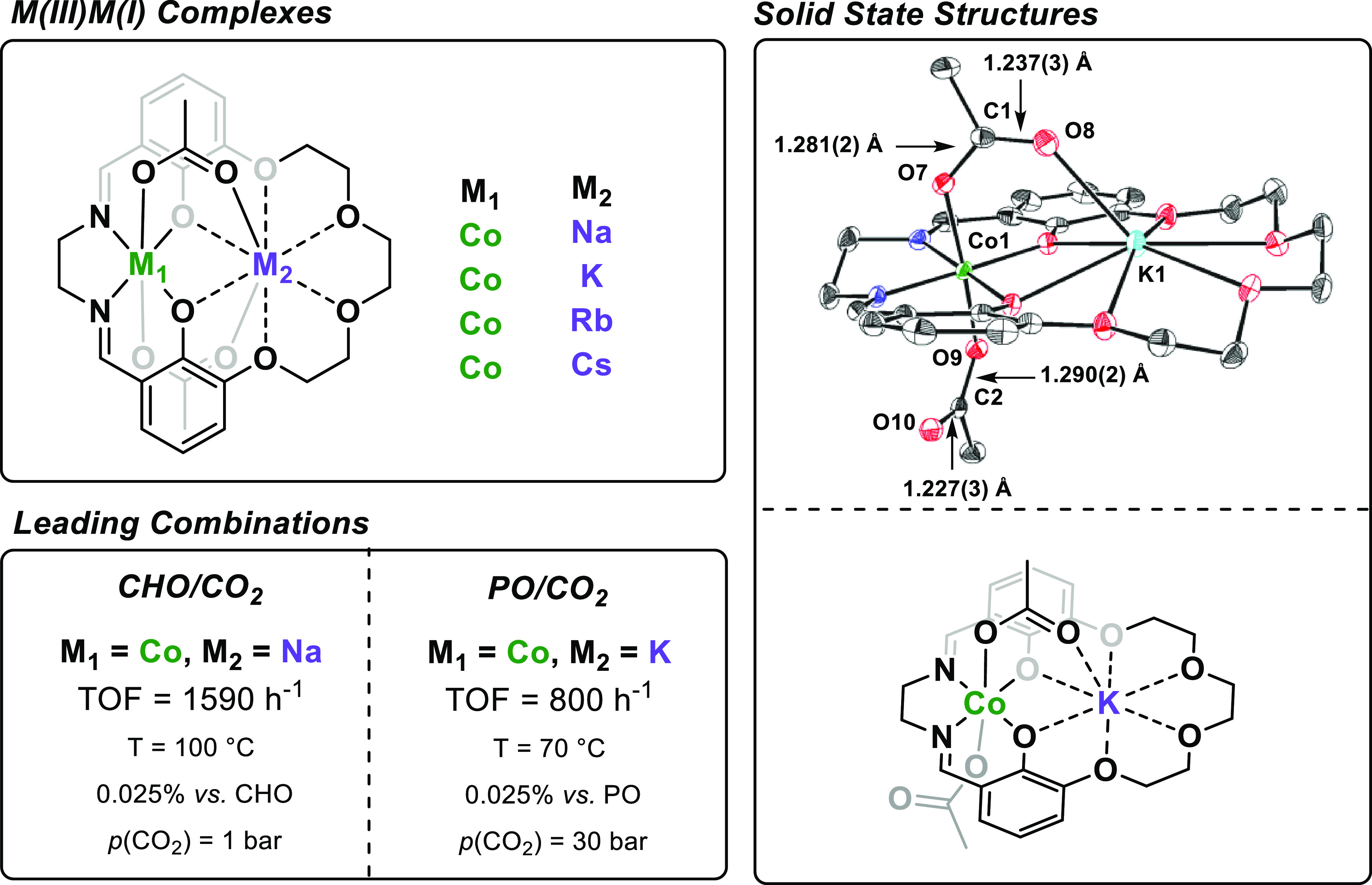
Heterodinuclear Co(III)M(I) catalysts, coordinated by **L2**, for epoxide/CO_2_ ROCOP (left). Solid-state structure of Co(III)K(I) with acetate bond lengths and representative structure showing anionic binding of acetates to Co(III) center (right).

Several research teams have investigated s-block heterodinuclear metal complexes of **L2**, particularly focusing on the influence of local electric fields on electron, proton, and hydrogen atom transfers.^40−43^ We selected **L2** for heterodinuclear polymerization catalysis using transition metals(II/III) and s-block metals(I/II) (Figure 4, left). Our prior work using **L1** showed that these metal combinations were ineffective, but the “crown ether” binding pocket in **L2** is much better suited to s-block metal coordination chemistry. First, **L2** complexes using four different metal combinations were investigated for CHO/CO_2_ ROCOP: Zn(II)Na(I), Mg(II)Na(I), Co(III)Na(I), and Zn(II)Mg(II).^44^ The Zn(II)Mg(II) complex was completely inactive, in contrast to the synergy observed using these metals with **L1** (vide supra).^35^ Mg(II)Na(I) also showed a low activity, but Zn(II)Na(I) showed promising performances (TOF = 29 h^–1^, PCHC selectivity = 94%, 1 bar CO_2_, 0.025 mol % catalyst). The best catalyst was Co(III)Na(I), which showed a very high activity (TOF = 1590 h^–1^, PCHC selectivity >99%, 100 °C, 1 bar CO_2_, 0.025 mol % catalyst); it is the most active low-pressure CHO/CO_2_ ROCOP catalyst reported to date.

Our group also investigated propene oxide (PO)/CO_2_ ROCOP, since the monomer is a commodity chemical, and poly(propene carbonate) is an elastomer/plastomer (*T*_g_ = 30–40 °C).^45^ PO/CO_2_ ROCOP is challenging because PO has a lower ring strain than CHO, which reduces the driving force for polycarbonate formation, and because it also shows a lower barrier to cyclic carbonate formation. As a result, catalytic activities and selectivities for PO/CO_2_ ROCOP are generally lower than for equivalent reactions using CHO, and many **L1** catalysts are inactive for PO/CO_2_ ROCOP.

In 2020, we reported the first heterodinuclear catalysts for PO/CO_2_ ROCOP.^46^ The complexes feature Co(III) coordinated within the Schiff-base pocket and a Group 1 metal bound by the ether groups.

Co(III)K(I) and Co(III)Na(I) both show “cobaltate” solid-state structures, i.e., where both the acetate ligands coordinate as anions to the Co(III) (Figure 4, right).^44,46^ The X-ray diffraction data show significantly longer acetate C–O bond lengths for the oxygen atoms bound to Co(III) (i.e., single bonds and O^–^ anionic donors) and shorter acetate C–O bond lengths for the oxygen atoms bound to K(I) or Na(I) centers (i.e., double bonds and O dative covalent donors). The cobaltate structures suggest that the Co(III) centers are strong Lewis acids and are important to understanding the polymerization mechanisms, for which DFT investigations in collaboration with Buchard and Phanopoulos are underway. Further, both complexes have intermetallic separations between 3–4 Å [M(I) = Na, 3.388 Å; K, 3.698 Å], similar to other highly active dinuclear catalysts we have reported (vide supra).

All the new heterodinuclear complexes are active catalysts for PO/CO_2_ ROCOP (Table 3). The Co(III)K(I) catalyst is the most active and selective and achieves a TOF of 800 h^–1^ at low catalyst loading (0.025 mol % catalyst, 30 bar CO_2_, 70 °C; Table 3, entry 2). Heterodinuclear catalysts applying the larger ionic radii of Rb(I) or Cs(I) were much less active than analogous Na(I) or K(I) complexes, perhaps due to “poor fit” of the s-block metal with the crown ether portion of **L2** (Table 3, entries 1–5). The exceptional performance of Co(III)K(I) likely arises from a beneficial combination of metal sizes, intermetallic communication, and coordination chemistry; it is noted that K(I) has the highest binding affinity for 18-crown-6 and thus may be the most compatible s-block metal for **L2**. The importance of using **L2** is demonstrated by the finding that a 1:1 mixture of [(salen)Co(III)X] complex and 18-crown-6/KI, reported earlier by Lu and co-workers, mostly formed cyclic carbonate (41% PPC selectivity) with much lower activity than Co(III)K(I) (Table 3, entry 6). This result highlights the importance of the ligand coordination chemistry and adjacency effects between the metals to maximize both polymerization selectivity and activity.

**Table 3:** Heterodinuclear PO/CO_2_ ROCOP catalysts using **L2**^a^

#	catalyst	time (h)	conv.^b^ (%)	CO_2_^c^ (%)	polym.^d^ (%)	TON^e^	TOF^f^ (h^–1^)	*M*_n_ [*Đ*]^g^ (g mol^–1^)
1	Co(III)Na(I)	5.0	15	>99	79	600	120	2300 [1.08]
2	Co(III)K(I)	4.0	34	>99	98	1360	340	5900 [1.10]
3^h^	Co(III)K(I)	1.4	28	>99	93	1120	800	5800 [1.07]
4	Co(III)Rb(I)	23	31	>99	91	1240	54	6500 [1.07]
5	Co(III)Cs(I)	23	27	>99	84	1080	57	5600 [1.08]
6^i^	[(salen)Co(2,4-DNP)] + 18C6/KI	3.0	27	>99	41	540	182	4700 [1.43]

a Reaction conditions: [Cat]/[CHD]/[PO] = 1:20:4000, neat epoxide, 20 bar CO_2_, 50 °C.^3^

b Conversion of epoxide, determined by ^1^H NMR.

c Selectivity for carbonate vs ether, determined by ^1^H NMR.

d Selectivity for polymer vs cyclic carbonate, determined by ^1^H NMR.

e Turnover number (TON) = total number of moles of epoxide consumed/mol of catalyst.

f Turnover frequency (TOF) = TON/time (hours).

g Number-average molecular weight [dispersity], determined by GPC.

h Reaction conditions: [Cat]/[CHD]/[PO] = 1:20:4000, neat epoxide, 30 bar CO_2_, 70 °C.

i Reaction conditions: [Cat]/[KI]/[PO] = 1:1:2000, 15 bar CO_2_, 25 °C.^47^

The Co(III)K(I) catalyst also showed excellent tolerance to copolymerizations conducted using CTA: high activity was maintained even when 250 equiv of diol (CHD) were added (vs catalyst). The use of diols controls the poly(propene carbonate) molar mass and selects for hydroxyl telechelic end-groups; at high loadings polyols are produced which may be useful for subsequent polyurethane manufacture. Co(III)K(I) can also be used with other epoxides, including cyclic (CHO, vinyl-CHO, and cyclopentene oxide) and acyclic (vinyl-PO, allyl glycidyl ether, and *tert*-butyl glycidyl ether) epoxides to form a range of different polycarbonates.

Kinetic analysis of the Co(III)K(I) catalyst shows a first-order dependence on catalyst and PO concentrations and a zero-order in carbon dioxide pressure (5–30 bar), indicative that epoxide ring-opening from a nucleophilic carbonate is the rate-determining step. The use of Eyring analysis gives a Δ*H*^‡^ of +13.4 kcal mol^–1^ and Δ*S*^‡^ of −26.9 cal mol^–1^ K^–1^, which results in a Δ*G*^‡^_323 K_ of +22.1 kcal mol^–1^. We are currently undertaking detailed computation and kinetic experiments in order to better understand the polymerization mechanism—such insights are critical to accelerating discovery of other heterodinuclear catalysts using these ancillary ligands. The catalysts will also be tested in various other polymerization catalyses including heterocycle ring-opening polymerization; heterocycle/heteroallene ROCOP; and switchable catalyses, which bridge both mechanisms.

### Epoxide/Anhydride ROCOP with **L3**

The success of heterodinuclear catalysis in yielding effective systems for epoxide/CO_2_ ROCOP naturally led to investigation into the related epoxide/anhydride ROCOP. Polyesters are important sustainable polymers since many monomers are, or could in future be, biobased; materials are recyclable and, after use, can be (bio)degraded to small molecules/monomers.^48^ Further, with the right catalysts, epoxide/anhydride ROCOP can be well controlled to produce semiaromatic, rigid, functionalized, and/or aliphatic polyesters; catalyst development is a priority. The two ROCOP reactions are similar but not identical, thereby meaning that catalysts must be considered and optimized for polyester production. For example, Al(III) catalysts typically underperform in CO_2_/epoxide ROCOP but many are excellent for anhydride/epoxide ROCOP.^49−51^ The difference is proposed to arise from a low barrier to Al-alkoxide bond dissociation that normally results in cyclic carbonate formation when carbon dioxide is applied, but since this side reaction is not prevalent when anhydrides are used, facilitates epoxide coordination and activation.^6,49^

**Figure 5:**
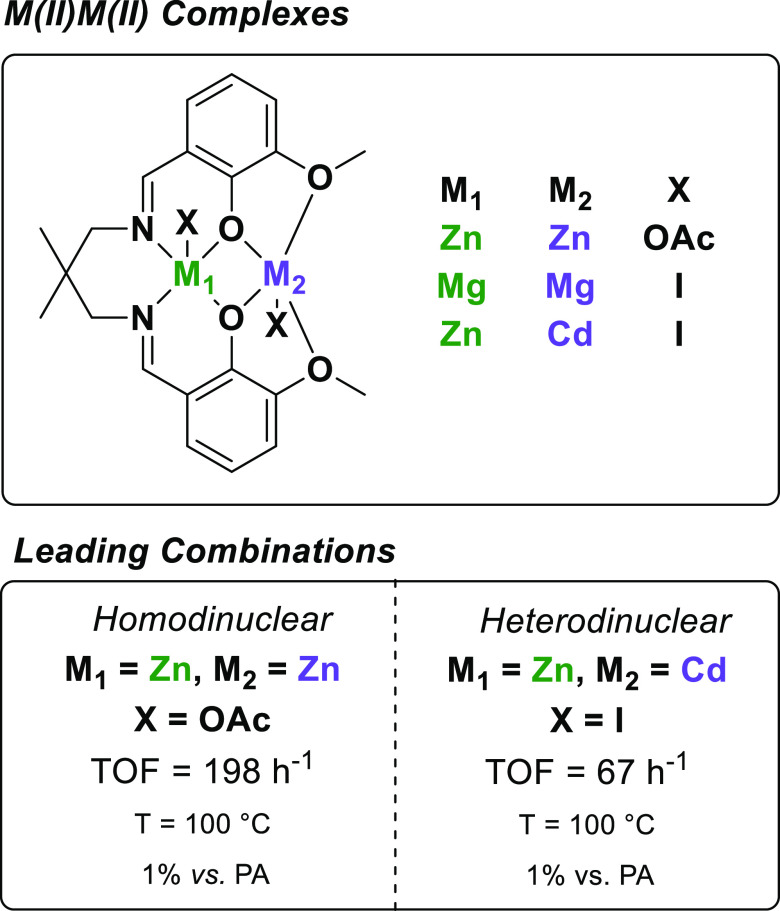
Dinuclear catalysts using **L3** for PA/CHO ROCOP.

In 2015, we demonstrated that **L1**Zn(II)Mg(II) was an effective catalyst for CHO/PA ROCOP, with the complex showing a TOF of 188 h^–1^ (1 mol % catalyst vs PA, 100 °C).^36^ The heterodinuclear complex outperforms the homodinuclear Zn(II)Zn(II) and Mg(II)Mg(II) congeners—the same heterodinuclear synergy as was observed for epoxide/CO_2_ ROCOP (vide supra).^52^ In the same year, we also reported a homodinuclear Zn(II)Zn(II) complex coordinated by Schiff base ligand **L3** that showed a higher activity for CHO/PA ROCOP (TOF = 198 h^–1^, 1 mol % catalyst vs PA, T = 100 °C) than **L1**Zn(II)Mg(II), despite being a homodinuclear complex (Figure 5).^53^ Hence, subsequent investigations using **L3** targeted heterodinuclear complexes.^54^ The heterodinuclear Zn(II)Mg(II) was not isolable because metal scrambling formed the Zn(II)Zn(II) and Mg(II)Mg(II) homodinuclear species, i.e., the heterodinuclear complex was not stable using this ligand.

Other heterodinuclear complexes using either Mg(II) or Zn(II) were successfully synthesized and were stable with respect to homodinuclear complexation. None of these heterodinuclear complexes, nor the homodinuclear Mg(II)Mg(II) complex, showed any higher activity than Zn(II)Zn(II). The most effective was Zn(II)Cd(II), which showed a moderate activity (TOF = 67 h^–1^, 1 mol % catalyst vs anhydride, 100 °C). Besides this lower catalytic activity [in comparison with Zn(II)Zn(II)], complexes containing heavy, expensive, and toxic metals such as Cd(II) are not ideal catalytic targets, and so further catalyst exploration was warranted.

**Figure 6:**
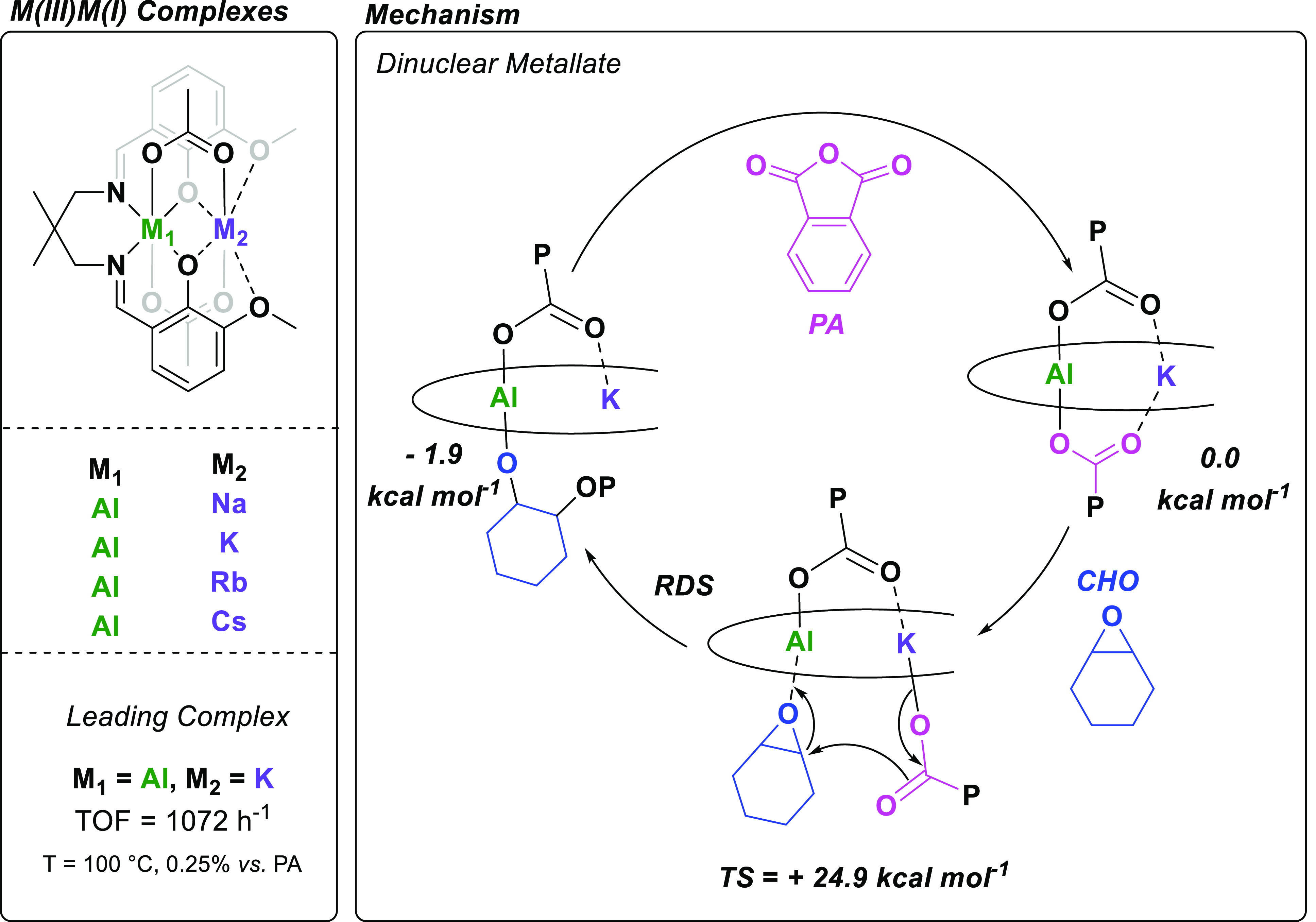
Heterodinuclear Al(III)M(I) catalysts coordinated by **L3** for epoxide/anhydride ROCOP (left). The proposed ROCOP mechanism is based on kinetic studies, solid-state structural data, and DFT calculations [theory level: ωb97xD/6-31+G(d,p)-H,C/6-311+G(d)-Al,K,O,N] (right).

In search for effective metal combinations, M(III)M′(I) heterodinuclear complexes were proposed. We had already investigated mononuclear Al(III) complexes coordinated by **L3**, which showed good epoxide/anhydride ROCOP activity but required a cocatalyst.^55,56^ In that work, only the Schiff-base (*N,N,O,O*)-groups were used to coordinate Al(III); hence, a logical next step was to investigate Al(III)M(I) complexes [M(I) = Na(I), K(I), Rb(I), Cs(I)] utilizing the ether-coordination pocket to coordinate the s-block metal (Figure 6, left).^4^

The resultant complexes were exceptionally active ROCOP catalysts, with the most effective being Al(III)K(I) and Al(III)Rb(I) with TOFs of 1072 h^–1^ and 1136 h^–1^, respectively (Table 4, entries 2 and 3). Al(III)Na(I) and Al(III)Cs(I), although slower than the best complexes, also displayed very high activity for this field of catalysis (Table 4, entries 1 and 4). All the catalysts were quantitative in their selectivity for polyester, forming no polyether linkages. Subsequent study was carried out utilizing the Al(III)K(I) complex because K is significantly lighter and more earth-abundant than Rb. The Al(III)K(I) catalyst showed high activity using other epoxides, including alkylene oxides, like PO, and anhydrides. This broad substrate scope allows it to furnish polyesters with a range of properties. It was also tolerant to very low catalyst loading and maintained equivalent activity at 0.005 mol % loading (vs CHO, i.e., [Cat]/[PA]/[CHO] = 1:4000:20000).

**Table 4:** Heterodinuclear PA/CHO ROCOP Catalysts Using **L3**^a^

#	catalyst	time (min)	conv.^b^ (%)^b^	polyester^c^ (%)	TON^d^	TOF^e^ (h^–1^)	*M*_n_ [*Đ*]^f^ (g mol^–1^)
1	Al(III)Na(I)	15	45	>99	180	720	9300 [1.06]
2	Al(III)K(I)	15	67	>99	268	1072	14300 [1.06]
3	Al(III)Rb(I)	15	71	>99	284	1136	14400 [1.06]
4	Al(III)Cs(I)	15	54	>99	216	875	10400 [1.05]

a Reaction conditions: [Cat]/[PA]/[CHO] = 1:400:2000, neat epoxide, 100 °C.

b Conversion of anhydride, determined by ^1^H NMR.

c Selectivity for polyester vs polyether, determined by ^1^H NMR.

d Turnover number (TON) = number of moles of anhydride consumed/number of moles of catalyst.

e Turnover frequency (TOF) = TON/time (hours).

f Number-average molecular weight [dispersity], determined by GPC.

Further investigations looked to develop an understanding of the role of the two metals during catalysis (Figure 6, right). Kinetic studies revealed a first-order rate dependence on both catalyst and epoxide concentration and a zero-order dependence on anhydride concentration. Hence, it was concluded that epoxide ring-opening is the rate-determining step. Since, in order to form ester linkages, epoxide ring-opening can only occur from a carboxylate chain end, the solid-state molecular structure of Al(III)K(I), which features two acetate coligands, is a useful model for the catalyst resting state. Analysis of the solid-state molecular structure of Al(III)K(I) revealed a 3.665 Å separation of the two metals, similar to both the highly active **L2**Co(III)K(I) and **L1**Mg(II)Co(II) complexes, and further demonstrates that intermetallic separation in the range of 3–4 Å is likely key in enabling intermetallic synergy. Like the **L2**Co(III)K(I) catalyst for CHO/CO_2_ ROCOP, it shows a “metalate” structure with both acetate groups coordinated as anions to the Al(III). Subsequent DFT calculations, conducted in collaboration with Buchard at the University of Bath, applied an Al(III)K(I) complex with benzoate coligands, which better modeled the propagating phthalate chain-end present in polymerizations. These calculations suggest that the Al(III) center binds and activates the epoxide, while the K(I) center coordinates the growing polymer chain through dative covalent bonds. This coordination ensures the growing chain is close to the active site and may facilitate monomer insertions (Figure 6, right). We have termed this pathway the “dinuclear metallate mechanism.”

There are some parallels between the proposed dinuclear metalate mechanism and the hypotheses for metal salen/cocatalyst systems.^56^ In both cases, the catalysts show metalate resting states and the polymer chain must undergo an initially unfavorable dissociation from M(III) to allow for subsequent epoxide coordination and activation. However, while salen-type catalysts require external cocatalysts [typically bis(triphenylphosphine)iminium chloride (PPNCl)] to aid the initial polymer chain dissociation, these new heterodinuclear complexes facilitate this process through the s-block M(I) center. The requirement for an external cocatalyst of metal-salen catalyst systems renders them inactive at moderate/low catalyst loadings, while heterodinuclear catalysts show excellent loading tolerance. Further, PPNX cocatalysts are significantly heavier than group 1 metals (e.g., [PPN]^+^ = 538.59 g mol^–1^, K^+^ = 39.1 g mol^–1^) and are typically expensive and may be corrosive to steel. Therefore, it is desirable to avoid their use.

## Conclusions and Outlook

**Figure 7:**
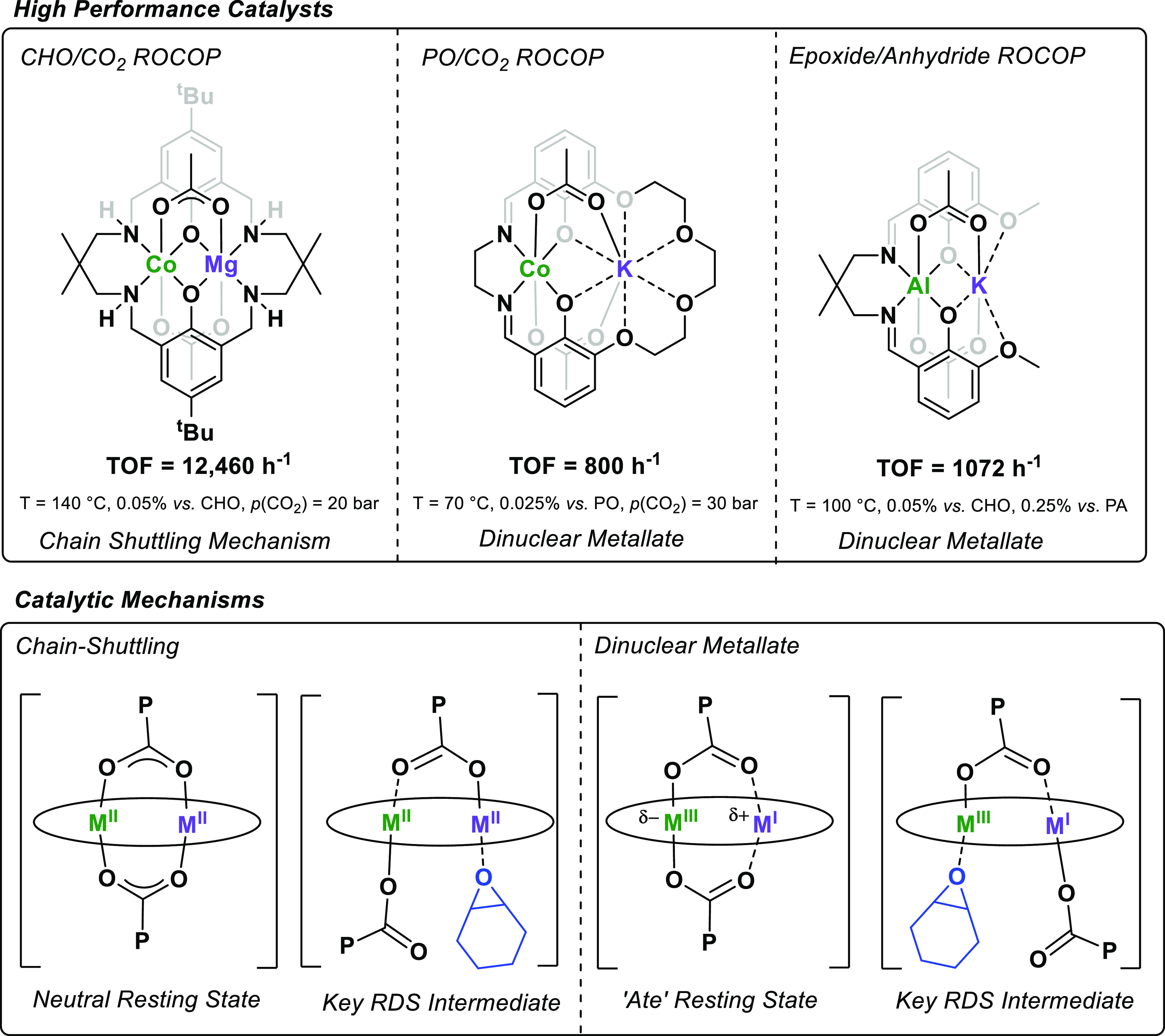
Leading heterodinuclear catalysts for CHO/CO_2_, PO/CO_2_, and CHO/PA ROCOP (top). Catalytic mechanisms for the different catalysts (bottom).

This Account has described some of our approaches to the discovery, development, and understanding of dinuclear catalysts for the ROCOP of epoxides with CO_2_ or anhydrides, with a particular focus on heterodinuclear synergic metal combinations. The best heterodinuclear catalysts show field-leading rates, high selectivity, excellent loading tolerance, and are effective using many different monomers (Figure 7, top panel). Nonetheless, the selection of the metal combination is critical, and not all heterodinuclear complexes are synergic. Catalyst design must correctly match the right metals with the most appropriate ancillary ligands; intermetallic separation, metallic electronic communication, donor atom chemistry, and the nature of the coligands are all important parameters underpinning effective heterometallic synergy.

The most effective polycarbonate catalysts utilize Co(II), Co(III), or Fe(III), with partner s-block metals such as Mg(II), Na(I), or K(I). The best polyester catalysts benefit from combining Al(III) with Group 1 metals. The use of earth-abundant, inexpensive, and nontoxic metals in catalysis, particularly Al(III), Fe(III), and Group 1 and 2 metals, is a significant advantage compared with many traditional catalysts, which typically apply transition metals and cocatalysts. In most cases, these earth-abundant metals show little-to-no activity by themselves; the heterodinuclear pairing is essential in yielding the high catalytic activities.

Our research implicates two different pathways by which these dinuclear catalysts may operate: (1) the chain shuttling and 2) the dinuclear metallate mechanisms (Figure 7, bottom panel). In both mechanisms, the metals’ roles are separated into epoxide coordination and activation and the provision of a reactive nucleophile. The Lewis acidic metal center activates the epoxide monomers, which is a crucial part of the rate-determining step. The second metal provides a reactive “nucleophile” and/or may also be important in (dative covalent) coordination of the growing polymer chain close to the metal-coordinated epoxide. It is important to fully investigate the catalyst’s structure, kinetics, and intermediates to understand which mechanism best describes performance. As a rule-of-thumb, where metals of different oxidation state/charge are applied, i.e., M(III)M(I) complexes, a dinuclear metalate reaction appears most likely. For a homodinuclear system, such as Zn(II)Zn(II), a chain shuttling mechanism is proposed. Heterodinuclear M(II)M(II) complexes could follow either mechanism.

Our future investigations of heterodinuclear polymerization catalysts will prioritise the use of inexpensive, abundant (easily extracted), and light elements. It is essential to identify, structurally characterize, and study the reactivity of the catalytic intermediates. We will continue to research these processes using in operando and in situ spectroscopies and a portfolio of analytical techniques. Considering the polymers produced using these catalyses, there has been excellent progress in delivery of low molar mass, hydroxyl end-capped polycarbonate/ester polyols. These oligomeric polyols are useful, and exploration of structure–activity relationships in polyurethane, surfactant, and resin/cross-linking applications are ongoing in our group. It is also possible to use the same catalysts and processes to make high molar mass polycarbonates and polyesters—this field of research is under-investigated and warrants greater future attention. One attraction of these heterodinuclear catalysts is their loading and monomer scope tolerance; thus, we will use them to study polymer viscoelastic, thermal, and mechanical properties.^57−60^ Catalyst development will focus upon monomers that are biorenewable and/or extracted as waste coproducts. Therefore, the integration of these polymerizations with carbon capture technologies is a priority.^61,62^
